# Gene and MicroRNA Transcriptome Analysis of Parkinson's Related LRRK2 Mouse Models

**DOI:** 10.1371/journal.pone.0085510

**Published:** 2014-01-10

**Authors:** Véronique Dorval, Wim Mandemakers, Francis Jolivette, Laetitia Coudert, Rachid Mazroui, Bart De Strooper, Sébastien S. Hébert

**Affiliations:** 1 Centre de recherche du CHU de Québec, CHUL, Québec, Québec, Canada; 2 Département de psychiatrie et de neurosciences, Université Laval, Québec, Québec, Canada; 3 Département de biologie moléculaire, biochimie médicale et pathologie, Université Laval, Québec, Québec, Canada; 4 Center for Human Genetics, K.U.Leuven, Leuven, Belgium; 5 Department of Molecular and Developmental Genetics, VIB, Leuven, Belgium; Ecole Polytechnique Federale de Lausanne (EPFL), Switzerland

## Abstract

Mutations in *leucine-rich repeat kinase 2* (LRRK2) are the most frequent cause of genetic Parkinson’s disease (PD). The biological function of LRRK2 and how mutations lead to disease remain poorly defined. It has been proposed that LRRK2 could function in gene transcription regulation; however, this issue remains controversial. Here, we investigated in parallel gene and microRNA (miRNA) transcriptome profiles of three different LRRK2 mouse models. Striatal tissue was isolated from adult LRRK2 knockout (KO) mice, as well as mice expressing human LRRK2 wildtype (hLRRK2-WT) or the PD-associated R1441G mutation (hLRRK2-R1441G). We identified a total of 761 genes and 24 miRNAs that were misregulated in the absence of LRRK2 when a false discovery rate of 0.2 was applied. Notably, most changes in gene expression were modest (i.e., <2 fold). By real-time quantitative RT-PCR, we confirmed the variations of selected genes (e.g., adra2, syt2, opalin) and miRNAs (e.g., miR-16, miR-25). Surprisingly, little or no changes in gene expression were observed in mice expressing hLRRK2-WT or hLRRK2-R1441G when compared to non-transgenic controls. Nevertheless, a number of miRNAs were misexpressed in these models. Bioinformatics analysis identified several miRNA-dependent and independent networks dysregulated in LRRK2-deficient mice, including PD-related pathways. These results suggest that brain LRRK2 plays an overall modest role in gene transcription regulation in mammals; however, these effects seem context and RNA type-dependent. Our data thus set the stage for future investigations regarding LRRK2 function in PD development.

## Introduction

PD is the most common neurodegenerative movement disorder, which affects about 1–2% of the population over 60 years of age [1]. The main clinical symptoms of PD include tremor, rigidity, slowness of movement and postural instability. At the histopathological level, PD is characterized by dopaminergic neuronal loss in the substantia nigra and striatum, combined with the formation of intracellular Lewy bodies in degenerating neurons [2]. Mutations in LRRK2 represent a large genetic component of both familial and sporadic PD [3], [4]. The *LRRK2* gene encodes a large (∼280 kDa) multidomain protein harbouring both GTPase and kinase activities (reviewed in [5]). Mutations in LRRK2 are clustered mainly around the central kinase (e.g., G2019S, I2020T) or GTPase (e.g., R1441G, R1441C, N1437H) domains. LRRK2 is proposed to function in neurite outgrowth [6], synaptic endocytosis [7], and autophagy [8]. Despite these advancements, the underlying mechanisms involved in LRRK2-mediated neurodegeneration remain poorly defined, particularly in the context of the adult mammalian brain.

Mounting evidence suggests that abnormal regulation of gene expression may contribute to PD pathogenesis (reviewed in [9]). Interestingly, LRRK2 modulation is associated with gene transcriptional changes. For instance, Häbig *et al.* reported significant changes in gene expression upon LRRK2 knockdown in human dopaminergic SH-SY5Y cells [10]. This group also identified a subset of genes misregulated in PD patients harbouring the LRRK2 G2019S mutation. In another study, Schulz *et al*. identified numerous alterations in mRNA abundance in LRRK2 haploinsufficient embryonic stem cells [11]. More recently, Nikonova described various changes in gene expression in LRRK2 KO mice [12]. These authors also reported opposite gene transcriptome profiles in LRRK2 G2019S transgenic (Tg) mice. Although many genes have been documented in these studies, little or no overlap was observed. In addition, statistical methods and cut-off criteria diverged considerably between research groups (discussed in [9]), making reliable conclusions difficult. One study actually reported no changes at all upon LRRK2 expression and/or in the presence of LRRK2 pathological mutations, in both cell lines and human tissues [13].

LRRK2 is proposed to interact with proteins implicated in gene expression regulation (reviewed in [14]). One of these candidates includes Argonaute 2 (Ago2), a component of the RNA-induced silencing complex (RISC) and an essential factor for miRNA function in mammals [15], [16]. Once incorporated into the Ago2/RISC complex, miRNAs function to promote mRNA degradation [8], translation inhibition [17], [18], or both [19], [20]. Each miRNA can bind to and regulate several (up to hundreds of) mRNA transcripts, thus potentially controlling several biological pathways. Interestingly, Gehrke *et al.* showed that Ago1 (the functional homologue of Ago2 in Drosophila) deficiency rescued LRRK2-mediated neurodegeneration in flies [16]. These effects were mediated by miR-184* and let-7, through the transcription factors *e2f1* and *dp*.

In the present study, we sought to analyze gene and miRNA expression patterns in various LRRK2 mouse models, with as purpose to better understand the role of mammalian LRRK2 in gene and, for the first time, miRNA expression regulation in the adult mouse brain. Bioinformatics analysis identified various mRNA:miRNA regulatory networks affected by LRRK2 deficiency. A different picture was observed in mice expressing human LRRK2, either wildtype or a PD-related mutant form. Indeed, no major changes in gene expression were noticeable, while a diverging set of miRNAs was affected. Most miRNA changes occurred in LRRK2 KO and hLRRK2-WT mice. Overall, these results provide a new glimpse into the role of mammalian LRRK2 in normal and PD-affected brain.

## Results

### Characterization of LRRK2 Mouse Models

**Figure 1:**
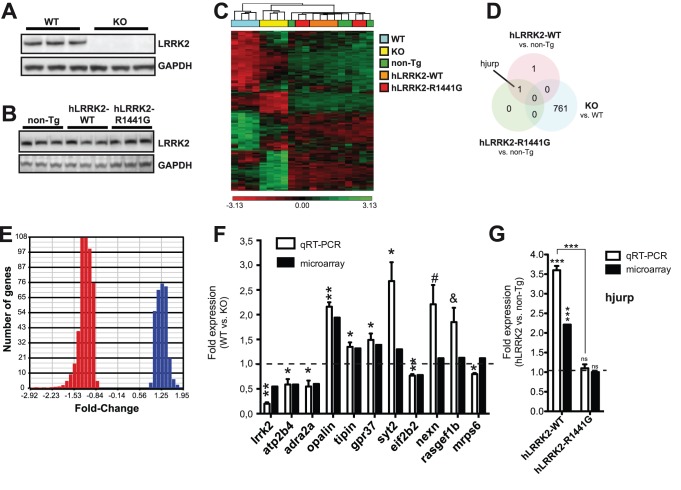
Gene microarray analysis of LRRK2 mice. (**A**) Representative western blot of endogenous striatal LRRK2 in 4 month-old wildtype and knockout mice. GAPDH was used as a loading control. Three samples (mice) are shown in each group. (**B**) Representative western blot of striatal LRRK2 in 4 month-old non-Tg, hLRRK2-WT and hLRRK2-R1441G transgenic mice. GAPDH was used as normalization control. Three samples (mice) are shown in each group. (**C**) Cluster analysis of significantly (FDR<0.2) misregulated genes (mRNA transcripts) in various LRRK2 mouse models. Note that both WT and KO mice cluster together. This is not the case for non-Tg and hLRRK2-R1441G mice. Results were generated using Partek Genomics Suite. (**D**) Venn diagram showing no overlap between significantly (FDR<0.2) misregulated genes in LRRK2 mice. One gene, *hjurp*, was specifically upregulated in hLRRK2-WT mice. (**E**) Histogram showing that >99% of misregulated transcripts has less than 2-fold difference in expression in LRKK2 KO mice when compared to littermate controls. (**F, G**) Validation of genes by real-time qRT-PCR. Statistical significance was determined by a Student unpaired t test (* = p<0.05, ** = p<0.01, *** = p<0.001, # p = 0.063, & p = 0.0566). Standard error of the mean (SEM) is shown.

LRRK2 is developmentally regulated in the mouse brain [21], to reach maximum levels at post-natal day 20 (P20) and subsequently maintained for up to 13 months (Fig. S1). Given these observations, we chose adult (4 month-old) mice for further analyses. At this age, no obvious phenotype was observed in all mouse models tested (see methods), which is consistent with previous observations [22]–[24]. Recently, it was shown that endogenous mouse LRRK2 was highly expressed in the striatum [25]. Western blot analysis using the MJFF2 antibody confirmed the absence of LRRK2 protein in striatal tissue isolated from LRRK2 KO mice when compared to wildtype littermate controls (Fig. 1A). A similar experiment was performed in mice expressing either hLRRK2-WT or hLRRK2-R1441G (Fig. 1B). Because expressed from a BAC construct, the LRRK2 human transgenes are driven by endogenous promoter and regulatory regions. As control for this group, we used non-transgenic (non-Tg) mice with a similar genetic background. No significant changes in total LRRK2 protein levels were noticeable in both hLRRK2-WT and hLRRK2-R1441G mice using the MJFF2 antibody. Similar results were obtained using the anti-LRRK2 UDD3 and N241A/34 antibodies, all of which recognize both mouse and human LRRK2 in similar extraction conditions (Figs. 1B and S2) [26]. Interestingly, a different picture was observed using anti-LRRK2 N231B/34, a high affinity antibody against human LRRK2 [26]. This latter antibody showed a 3–4 fold increase in human LRRK2 expression in LRRK2-R1441G mice when compared to hLRRK2-WT mice (Fig. S2B). Thus, human LRRK2 is more abundant in hLRRK2-R1441G mice, while total LRRK2 protein levels remain unchanged amongst non-Tg and Tg mice.

### Analysis of Gene Expression Profiles in LRRK2 KO and Tg Mice

We next performed genome-wide microarrays (Affymetrix GeneChip Gene) on striatal tissue isolated from LRRK2 KO mice and littermate controls (n = 4 per group). We identified a total of 761 genes that were misregulated in the absence of LRRK2 when a false discovery rate (FDR) of 0.2 was applied (Fig. 1C, 1D and Table S1). From those transcripts, 38% and 62% were downregulated and upregulated, respectively. Note that most (710/761) gene expression changes were below 1.5 fold (Fig. 1E). It should be cautioned, however, that LRRK2-negative cells (e.g., oligodendrocytes or astrocytes [25]) might also contribute to the overall weak probe signals on the microarray chips. By qRT-PCR, we validated 11 misregulated genes selected based on significant P values and fold changes (Fig. 1F and Table S2). These genes included *lrrk2* itself (p = 0.002, 0.2 fold), *syt2* (p = 0.032, 2.7 fold), *opalin* (p = 0.005, 1.9 fold) and *eif2b2* (p = 0.011, 0.8 fold). Complete fold changes, accession numbers and oligonucleotide sequences, when available, are listed in Table S2.

We next aimed to determine whether PD-related mutations in LRRK2 influenced gene expression patterns. To this end, we analyzed gene transcriptome profiles of hLRRK2-WT and hLRRK2-R1441G mice (n = 4 per group). As before, we isolated the striatal tissue from 4 month-old animals. When compared to non-Tg controls, no changes in gene expression were observed in hLRRK2-R1441G mutant mice when a FDR of 0.2 was applied, whereas only two genes reached significance in hLRRK2-WT mice (Fig. 1C, D and Table S1). Lowering stringency levels (FDR up to 50%) did not considerably increase the number of misregulated transcripts in these models (Table S3). By qRT-PCR, we confirmed the change of *hjurp* mRNA levels (p<0.001, 3.6 fold) in hLRRK2-WT mice when compared to non-Tg and hLRRK2-R1441G mice (Fig. 1G). These observations demonstrate that 1) the expression of human LRRK2 has little influence on gene expression patterns *in vivo* in the Tg mice, and 2) the PD-associated hLRRK2 R1441G mutation is a loss of function in this context, at least with regard to *hjurp* expression regulation. Interestingly, *hjurp* encodes a histone chaperone that contributes to high-fidelity chromosome segregation during cell division. Abnormal regulation of chromosome segregation has previously been linked to neurodegenerative disorders such as Alzheimer’s disease [27].

### Analysis of miRNA Expression Profiles in LRRK2 KO and Tg Mice

**Figure 2:**
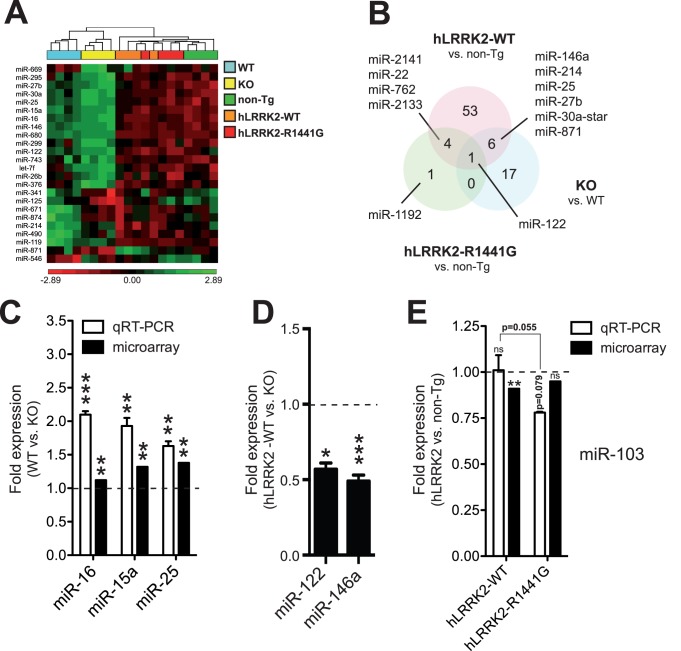
miRNA microarray analysis of LRRK2 mice. (**A**) Cluster analysis of significantly (FDR<0.2) misregulated mature miRNAs in various LRRK2 mouse models. Results were generated using Partek Genomics Suite. (**B**) Venn diagram showing variable overlap between significantly (FDR<0.2) misregulated miRNAs in LRRK2 mice. (**C, D and E**) Validation of miRNAs by real-time qRT-PCR, as indicated. Statistical significance was determined by a Student unpaired t test (* = p<0.05, ** = p<0.01, *** = p<0.001). Standard error of the mean (SEM) is shown.

miRNA microarray analysis (Affymetrix GeneChip miRNA) was performed on all mouse models tested above. For comparative reasons, we used the same RNA samples (n = 4 per group) used to perform the gene expression analyses. These experiments identified 24 mature miRNAs that were misregulated in LRRK2 KO mice when a FDR of 0.2 was used (Fig. 2A, B and Table S4). By qRT-PCR, we confirmed significant changes in 3 miRNAs in LRRK2-deficient mice when compared to controls, including miR-16 (p<0.0003, 2.1 fold), miR-15a (p = 0.0128, 1.9 fold), and miR-25 (p = 0.0037, 1.6 fold) (Fig. 2C). These miRNAs were selected based on P values and biological pathways of interest (see below). Using similar stringency, we also identified 64 and 6 miRNAs that were changed in hLRRK2-WT and hLRRK2-R1441G mice, respectively, when compared to the non-Tg controls. Interestingly, some overlap was observed between LRRK2 KO and hLRRK2-WT mice following normalization to respective controls (Fig. 2B). It should be noticed that a direct comparison between mice groups (i.e., LRRK2 KO vs. hLRRK2 Tg mice) cannot be performed since bred on different backgrounds (see Methods). We could confirm miR-122 (p = 0.0290, 0.56 fold) and miR-146a (p = 0.0009, 0.49 fold) changes between LRRK2 KO and hLRRK2-WT mice using RNU19 as normalization control (Fig. 2D).

Interestingly, miR-103 was specifically affected in hLRRK2-R1441G mice (Table S4). As seen with the mRNA transcripts, most alterations were modest (<2–3 fold). We validated changes in miR-103 levels by qRT-PCR (p = 0.055, 0.79 fold change) (Fig. 2E). Taken together, these results suggest that LRRK2 influences miRNA output differently according to LRKK2 species and the PD-related mutation. No direct correlation between (human) LRRK2 expression levels and number of misregulated transcripts are evident. Moreover, LRRK2 function is associated with RNA-type dependent alterations, that is, mRNA vs. miRNA.

### Identification of mRNA:miRNA Networks in the Absence of LRRK2

Microarray data mining using the DAVID (see methods) identified several biological networks affected in LRRK2 KO mice (Table S5). These included gene ontology (GO) terms such as *cytoplasmic membrane-bounded vesicle*, *ATPase activity,* and *synapse part*. These in silico analyses are consistent with previous studies linking LRRK2 to, among other functions, synaptic endocytosis and mitochondria [7], [28].

We also used the Ingenuity Pathway Analysis (IPA) tool to search for molecular pathways associated with LRRK2 loss in the mammalian brain. Biological functions, canonical pathways and gene networks generated by IPA are provided in Table S5. High-ranking pathways included *cellular function and maintenance* as well as *Parkinson’s signaling*. Interestingly, the highest-ranking predicted *upstream regulator* of LRRK2-dependent pathways was MAPT, the gene encoding tau protein (Table S5 and Fig. S5). Recent evidence supports a functional role between LRRK2 and tau phosphorylation, through either a direct interaction between both proteins or via GSK3β [29], [30]. Together, these results highlight the potential importance of LRRK2 in regulating both biologically and pathologically relevant pathways, perhaps through direct and indirect mechanisms.

**Figure 3:**
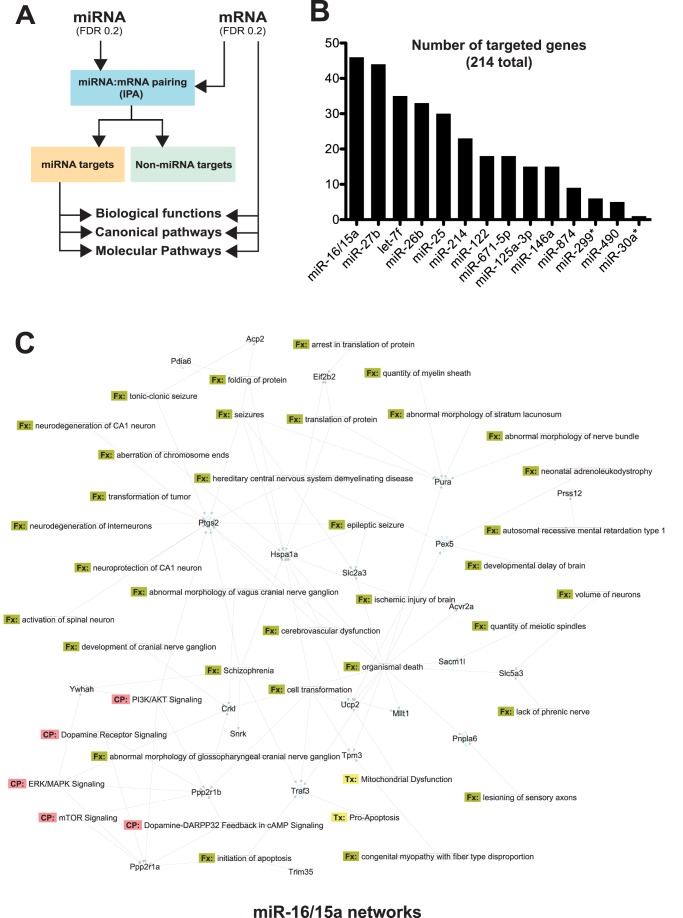
Bioinformatics analysis of LRRK2-dependent pathways. (**A**) Schematic overview of mRNA:miRNA pairing performed using the IPA software. This analysis allows the discrimination between potential miRNA targets and non-miRNA targets. The generated lists of genes are then processed for biological and functional significance. (**B**) Number of IPA-generated mRNA:miRNA pairings. Thirteen misregulated miRNAs (from a total of 24) are predicted to regulate 214 genes (from a total of 671). The top-ranking miRNAs are miR-16 and miR-15a, which harbour the same seed sequence (GCTGCT), and thus functional mRNA binding site. Changes in miR-16/15a levels were confirmed by qRT-PCR. (**C**) Shown here are statistically significant (P<0.05) IPA-generated networks. Both upregulated and downregulated genes predicted to be regulated by miR-16/15a were included in the analysis. Highlighted pathways are relevant for brain function and disease. The image was generated using the IPA software.

The identification of both gene and miRNA variations in LRRK2 KO mice prompted us to analyze in more detail potential mRNA:miRNA regulatory networks (Fig. 3A). Only opposite correlations (i.e., increased miRNAs associated with decreased mRNAs and *vice versa*) were included in this analysis. Bioinformatics analysis using IPA identified a total of 214 mRNA:miRNA pairings (Fig. 3B), suggesting that up to 1/3 of mRNAs are prone to miRNA regulation in this model. Highest-ranking miRNAs included miR-16/15a (46 targets), miR-27b (44 targets), let-7f (35 targets), miR-26b (33 targets), and miR-25 (30 targets). Of note, most miRNAs had overlapping target mRNA transcripts (data not shown), consistent with the notion of cooperative regulation of mRNA abundance by multiple miRNAs [31]. When analysed altogether, miRNA-dependent pathways were associated with biologically important pathways and networks.

Detailed analysis of miR-16/15-regulated genes (ranked first) showed an association with biological terms such as *neurological disease* (P<0.001), *cell death and survival* (P<0.001), and *PIK3/Akt signaling* (P<0.001) (Fig. 3C). These predictions are in agreement with the literature suggesting a role for miR-16/15 in cell survival [32], [33]. In addition, one gene which has been found in the microarrays and further validated by qRT-PCR was present in this network. That is the eukaryotic translation initiation factor 2B (Eif2b2). Furthermore, we highlighted potential pathologically-relevant networks (genes) regulated by miR-16/15. Notably, the network includes functions such as translation of proteins, mTOR and PI3K/Akt signaling. The list also included cancer-related genes. Recent association studies have linked LRRK2 with specific types of cancer, but like neurodegenerative diseases, the underlying mechanisms remain elusive (reviewed in [34]).

### Biochemical and Functional Analysis of Ago2 in LRRK2 Mice

**Figure 4:**
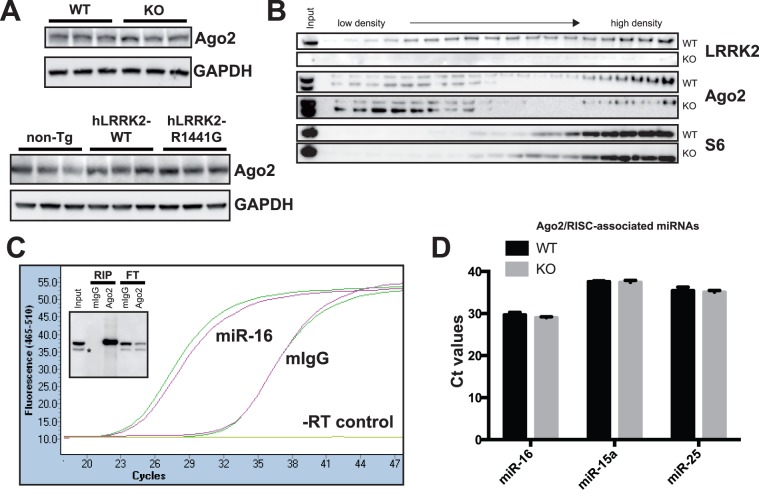
Biochemical and functional analysis of Ago2 in LRRK2 mice. (**A**) Representative western blot analysis showing that Ago2 protein levels are not affected in the presence or absence of LRRK2 (top panel). Overexpression of human LRRK2-WT or harbouring the human PD mutation R1441G has no effect on Ago2 levels either, when compared to non-transgenic littermates (bottom panel). (**B**) Polysomes fractionation of LRRK2-deficient mouse brain, compared to wild-type brain. No change in Ago2 or the ribosomal protein S6 distribution was observed by the absence of LRRK2. (**C**) RIP-Ago2 assay of wild-type mouse brain. A representative amplification curve of miR-16 by real-time qRT-PCR shows a significant enrichment (∼500 fold) of this miRNA pulled down by RIP-Ago2. The insert demonstrates the efficiency of the immunoprecipitation (Ago2 vs. mouse IgGs). The flow-through (FT) shows the concomitant decrease of Ago2 in the post-RIP lysate. The “*” sign is Radixin, a well known non-specific protein when using the Ago2 (2A8) antibody [47]. Of note, Radixin is not immunoprecipitated by Ago2 (2A8). (**D**) Histogram of RIP miRNA pulled down in the presence or absence of LRRK2. In each case (n = 3), there is no difference in Ct values (qRT-PCR). LRRK2 deficiency does not affect RIP-Ago2 RNAs or miRNAs immunoprecipitation. Standard error of the mean (SEM) is shown.

Given that miRNAs were consistently affected in the LRRK2 mouse models, we next thought to analyse in more detail the relationship between LRRK2 and Ago2 in the mammalian brain. We first characterized the effect of LRRK2 deficiency, as well as the expression of human LRRK2 species, on endogenous Ago2 levels. As shown in Figure 4A, no change in Ago2 expression was observed in all mouse models tested. Previous studies in flies have suggested that LRRK2 could bind directly to Ago2 [16]. However, this interaction could not be validated under physiological conditions in the mouse brain using reciprocal co-immunoprecipitations (Fig. S3 and data not shown). Gehrke *et al.* reported that LRRK2 co-localized with Ago1 in polysomes, an active site of miRNA function and protein translation [35], [36]. In the mouse brain, and as expected, Ago2, co-localized mainly with the polysome marker FMRP (Fig. S4) [37]. Subsequent detailed analysis demonstrated that LRRK2 and Ago2 co-localized partly in free ribonucleoprotein proteins (RNP) fractions (Fig. 4B). Here, the 40S ribosomal protein S6 was used as marker for monosomes. Notably, the subcellular distribution of Ago2 was unaffected upon LRRK2 deficiency.

We finally sought to determine whether Ago2 function, that is the binding and the maturation of miRNAs within the RISC complex, was affected by LRRK2 deficiency. To this end, we performed Ago2-RIP experiments [38]. We first confirmed the efficiency of Ago2 immunoprecipitation under our *in vivo* conditions (Fig. 4C, inner panel). As shown in Figure 4C, a strong enrichment (lower Ct values) in mature miRNA levels was observed in RIP-Ago2 immunoprecipitations when compared to controls. Here, a representative qRT-PCR using miR-16 is shown. Ago2/RISC-bound RNA was subsequently analysed by miRNA microarrays (Affymetrix GeneChip miRNA) (n = 2 per group). In contrast to our previous results using total RNA (Fig. 2A and 2B), we observed no significant changes in miRNA expression levels in LRRK2 KO mice when a FDR of 0.2 was applied (data not shown). These observations were validated by qRT-PCR on “positive” miRNAs (i.e., miR-16, miR-15a, and miR-25) (Fig. 4D) in an independent set of animals (n = 3 per group). Taken together, these results suggest that Ago2 function, as defined above, is not directly affected by LRRK2 deficiency. These observations are consistent with our fractionation and co-immunoprecipitation results showing dissociation between Ago2/miRNA localization (and thus function) and LRRK2 in the mammalian brain.

## Discussion

The main goal of this study was to provide primary information on transcriptional changes dependent on LRRK2 in the adult mammalian brain, with as purpose to contribute to the debate. In addition to knockout mice, we used two additional models expressing human LRRK2, bearing or not the pathological PD mutation R1441G. The results identified several mRNA transcripts affected by LRRK2 deficiency in the striatum, most of which seem biologically important. Our results tend to show, however, that human LRRK2, including a PD pathological form, does not substantially affect mRNA levels in adults. On the other hand, a number of miRNAs were misregulated in all mouse models, suggesting an RNA-type dependent regulation by LRRK2. As of yet, it remains unclear how mammalian LRRK2 mediates the effects on gene and miRNA expression.

Recently, Nikonova *et al.* identified a number of misregulated genes (up to ∼800) in LRRK2 KO and human LRRK2 G2019S mice [12]. The authors reported opposite changes in gene expression patterns between LRRK2 KO and LRRK2-PD-related mice. It is important to mention that such direct comparison between our mouse models could not be performed because bred on different genetic backgrounds. Nevertheless, and consistent with the results presented herein, they identified several biological pathways dependent on LRRK2 activity. Despite this, no clear overlap was observed between their microarray results (LRRK2 G2019S vs. KO) and ours (LRRK2 KO vs. wildtype). From the 22 genes differentially expressed in the Nikonova *et al.* study, only two genes (Spef2 and Cdkn1a) and four ribosomal proteins (RPL10a, RPL35a, RPS2 and RPS28) overlapped with our significant list of genes in the LRRK2 KO mice. Although unexpected, a number of reasons might explain the lack of overlapping results. First, mouse ages were not disclosed in the Nikonova *et al.* study. We cannot exclude at this stage of investigation age-dependent effects of LRRK2 on the transcriptome. Second, a different LRRK2 mutant was studied (G2019S is located in the kinase domain, whereas R144G is found in the GTPase domain). On this line of thought, Nikonova *et al.* did not use Tg mice expressing human LRRK2 wildtype as controls. Third, basal mRNA expression profiles are known to vary among mouse strains, especially within the brain [39]. Finally, and importantly, their study was more permissive, with estimated false discovery rates achieving 50%, and in some cases exceeding 80%. Nevertheless, our results are consistent with the concept that LRRK2 activity is important for gene transcription regulation in mice. The precise number and identity of genes regulated by LRRK2 *in vivo*, either by mouse or human variants, remains to be fully determined.

Since LRRK2 KO mice displayed both mRNA and miRNA changes, it was possible to perform detailed analysis of affected biological pathways using bioinformatics. This was exemplified by the generation of networks related to the miRNA family involved in the regulation of the highest number of genes, miR-16/15. These two miRNAs share the same seed sequence (GCTGCT), potentially regulating the same mRNA targets. This miRNA family is related to various conditions such as neurodegeneration and anxiety disorders [32], [40], [41]. miR-16/15 are also involved with mitochondrial dysfunction and apoptosis [33], [42]. Interestingly, PI3K/Akt and mTOR signaling, involved in translational control and protein output, are among the pathways potentially regulated by miR-16/15. It would thus be interesting to investigate the role of LRRK2 in protein translation regulation, for instance by performing proteomic studies. On this line of thought, the elongation factor EiF2B2, which was downregulated in the LRRK2 KO mice, is involved in protein translation regulation. Interestingly, a genome-wide analysis of a family with autosomal-dominant parkinsonism has implicated several missense mutations in the translation elongation factor EiF4G1 [43]. It should be emphasized, however, that LRRK2 was little or not present in active translation sites in the mouse brain. Whether LRRK2 plays an indirect (e.g., substrate phosphorylation) and/or transitory role in gene expression (and/or translation) regulation remains to be determined.

Our microarray analysis identified miR-103 to be specifically misregulated in hLRRK2-R1441G mice. This miRNA belongs to the miR-16/15 superfamily [41] and is predicted to regulate a large number of mRNA transcripts (476 conserved targets according to TargetScan.org). However, since no complementary changes in mRNA expression were identified in the LRRK2 mutant mice, it is difficult to predict the role of miR-103 in this mouse model and in the context of PD. Nevertheless, these results suggest that LRRK2 influences RNA types (mRNA vs. miRNA) differently according to LRKK2 species and PD pathological mutations. Based on bioinformatics predictions, no miR-103 target site is present in the 3′UTR of *hjurp* (data not shown) that is downregulated in the hLRRK2-R1441G mice, suggesting that this gene is not directly regulated by this miRNA. It would thus be relevant to identify and validate proteins regulated by miR-103 in the mouse brain, in both normal and pathological conditions.

Based on observations by Gehrke *et al.* in flies [16], we explored the potential relationship between LRRK2 and Argonaute in mice. We focused on Ago2 since being the sole mediator of miRNA function in mammals. Our combination of expression, immunoprecipitation, fractionation and RIP assays strongly suggest that LRRK2 is dispensable for Ago2/RISC function in the mouse brain. However, it should be kept in mind that our study does directly address the role of LRRK2 in miRNA-mediated protein translation, as suggested by Gehrke *et al.* These authors showed that miRNA levels were not affected *per se* by LRRK2 activity, a hypothesis not entirely discordant with our results. Notably, our microarray analyses identified no significant changes in miRNA precursor (pre-miR) levels in the hLRRK2 Tg models (data not shown), suggesting that miRNA maturation, in addition to loading miRNAs into the RISC complex, is unaffected by LRRK2 activity. The results presented herein nevertheless point to a context and likely species-dependent function of LRRK2, where results obtained from cell lines or invertebrates cannot be easily extrapolated to mammals *in vivo*. This is in line with our observation that in LRRK2 mice, there is no change in effector genes such as *e2f1* and *dp1*, previously identified by Gehrke *et al.* in flies (Table S1 and data not shown).

Another potential player involved in LRRK2-mediated gene/miRNA expression regulation is 4EBP1 (reviewed in [9]). However, our preliminary observations point to no changes in 4EBP1 expression, phosphorylation, and localization in both LRRK2 KO and hLRRK2-R1441G mice (data not shown), consistent with recent observations [44]. Clearly, further experiments are required to fully understand the role of LRRK2 and its putative binding partners in transcription regulation in the adult mammalian brain.

In conclusion, this is the first study, to our knowledge, that investigated in parallel mRNA and miRNA transcriptomes of 3 different but complementary LRRK2 mouse models. Our results demonstrate that LRRK2 plays a modest role in gene and miRNA expression regulation, consistent with previous reports in cells, mice and humans. We hope our combination of microarrays, qRT-PCR, bioinformatics, RIP and biochemical experiments will stimulate follow-up studies addressing the role of LRRK2 in normal and pathological conditions. Important issues that remain include the identification of additional LRRK2 partners and/or substrates, to extend its role in the context of aging, miRNA-mediated pathways, translation, and disease.

## Materials and Methods

### Antibodies

Rabbit monoclonal anti-LRRK2 MJFF-2 (clone c41-2) and UDD3 were from Epitomics (Burlingame, USA). Mouse monoclonal anti-LRRK2 N241A/34 and N231B/34 were from NeuroMab (Davis, USA). Mouse anti-panAgo2 (2A8) and rabbit anti-Ago2 (C34C6) were from Millipore (Billerica, USA) and Cell Signaling (Denver, USA), respectively. Mouse anti-S6 (5G10, Cell Signaling), mouse anti-GAPDH (MAB374, Millipore) were also used. HRP-conjugated secondary antibodies (mouse and rabbit) as well as control immunoglobulins were obtained from Jackson ImmunoResearch Laboratories (Baltimore, USA).

### Animals and Phenotypes

Details can be found elsewhere [25]. Briefly, mice were housed under specific pathogen-free conditions and were used in accordance with the University of Leuven Animal Ethics Committee. LRRK2 knockout (B6-LRRK2 <tm1149.2Arte>) mice (Taconic, Netherlands) were generated by deletion of the genomic region covering exon 41 through 47. Heterozygous LRRK2 KO mice were mated, and wildtype and homozygous littermate offspring were used for analysis at 4 months of age. LRRK2 KO mice are viable, live to adulthood and have no major abnormalities in dopaminergic neurons [23].

hLRRK2-WT (FVB/N-Tg(LRRK2)1Cjli/J) BAC-transgenic mice and hLRRK2-R1441G (FVB/N-Tg(LRRK2*R1441G)135Cjli/J) BAC-transgenic mice [22] were obtained from Jackson Laboratories, and bred with non transgenic FVB/N mice. Tg mice were kept at hemizygous state. hLRRK2-WT and hLRRK2-R1441G and non-transgenic littermates were used for analysis at 4 months of age. Mutant mice are viable and fertile, and showed no gross morphological and behavioural abnormalities (data not shown), consistent with previous results [22]–[24]. Sacrifices were performed by decapitation without anaesthesia. Tissues were isolated and snap frozen in liquid nitrogen until use.

### RNA and Protein Extraction

Total RNA was extracted using Trizol reagent (Invitrogen), according to manufacturer’s protocol. Chloroform was added and RNA, present in the aqueous phase following high speed centrifugation is next precipitated with cold isopropanol. Extracted RNA quality was assessed by Nanoquant (Tecan Infinite F200) and Bioanalyzer (Agilent technologies). An RNA integrity number (RIN) above 8 was used in all experiments. Notably, unless otherwise stated, the same RNA samples were used in all subsequent experiments. Proteins were purified using cell lysis buffer (50 mM Tris-HCl pH 7.4, 1%NP-40, 150 mM NaCl, 1 mM EDTA, 1 mM PMSF, 100 mM Na_3_VO_4_, 100 mM NaF and protease inhibitors). Proteins were separated by electrophoresis (Bis-Tris Nupage gels), transfered onto nitrocellulose and incubated with antibodies, as described. Positive bands were visualized by chemiluminescence using ECL according to manufacturer’s instructions. The image analyzer ImageQuant LAS4000 (GE Healthcare Bio-Sciences) was used to acquire images.

### RNA-binding Protein Immunoprecipitation (RIP)

The RIP protocol was performed as described previously [38]. Anti-Ago2 (2A8) and control mouse IgGs were coupled to protein G sepharose (GE Healthcare Bio-science). Tissues were homogenized in a lysis buffer (25 mM Tris-HCl pH8, 150 mM NaCl, 2 mM MgCl_2_, 0.5% Triton X-100, 5 mM DTT, 250 U/ml RNasin and protease inhibitors). Proteins were transferred to a clean tube after high speed centrifugation. Total lysate was pre-cleared by incubating with protein G alone and then separated into two fractions. These were incubated with either the antibody (Ago2) or control IgG-coupled beads. Following washes (high salt buffer = lysis buffer at 900 mM NaCl and low Triton X-100 buffer = lysis buffer at 0,05% Triton X-100), proteins, including RNA-binding proteins, were eluted with sample buffer. Immunoprecipitated RNAs were extracted directly from the beads using Trizol (Invitrogen), as described above. miRNA was then subjected to miRNA microarray analysis and, together with mRNAs, subjected to qRT-PCR analysis. Following the immunoprecipitation, the protein fraction was subjected to Western blot analysis (anti-Ago2 C34C6) in order to visualize the efficiency of Ago2 immunoprecipitation.

### Polysome Preparation

P30 mouse brains were homogenized in extraction buffer (20 mM Tris pH7.4, 1.25 mM MgCl_2_, 150 mM NaCl, 1 mM DTT, 1% Triton X-100), as recently described [45]. The cleared lysate was then deposited on top of a 50% sucrose fraction for pre-clear by centrifugation (35000 rpm for 2.5 h at 4°C). The pellet was resuspended in extraction buffer, layered on top of a discontinuous sucrose gradient (10–50%) and centrifuged at 35000 rpm for 2.5 h at 4°C. Equal fractions were collected from the top and mixed with protein loading sample buffer. Proteins were then subjected to Western blot analysis, as described above.

### Microarrays and Data Analysis

Microarray analyses were carried out as before [31] using Mouse Gene 1.0 ST and miRNA (v1 or v2) microarrays (Affymetrix). Bioinformatics analysis was performed using the Partek Genomics Suite software (http://www.partek.com/partekgs).

### Quantitative Real-time RT-PCR

cDNA synthesis from extracted mRNA (see above) was made using the Iscript synthesis kit, according to the manufacturer’s instructions (Bio-Rad, Mississauga, Canada). cDNA was then measure by qPCR (LightCycler 480 II, Roche) using SsoFast supermix (Bio-Rad). miRNA quantifications used the Taqman technology (Universal Taqman mastermix, Applied Biosystem, Burlington, Canada). All quantifications were performed as described before [31], [46]. The geometric mean of the housekeeping genes GAPDH and RPL32 was used as mRNA normalization controls. The small nucleolar gene RNU19 was used as miRNA normalization control. Relative expression of genes and miRNAs was calculated using the comparative CT (ΔΔCt) method.

### Ingenuity Pathway Analysis (IPA)

The list of significant genes identified by Partek Genomics Suite, containing Affymetrix probe set IDs, fold changes and p values, were uploaded into the Ingenuity Pathway Analysis (IPA) tool (www.ingenuity.com). Detailed analysis of focus genes was used for generating biological networks.

### Gene Ontology (GO) Analysis

GO term analysis of misregulated genes (FDR<0.20) was performed using the Database for Annotation, Visualization and Integrated Discovery (DAVID) version 6.7 (http://david.abcc.ncifcrf.gov/).

### Statistical Analysis

Statistical tests were performed using the GraphPad Prism version 6.0 software. Statistical significance (p<0,05) was determined using an unpaired Student’s t-test. Given unequal variances, a Welch’s correction test was applied.

## Supporting Information

Figure S1 LRRK2 levels during mouse development. Representative western blot analysis of brain LRRK2 expression at different time-points, from embryonic day 18 (E18) to 13 months of age. Tubulin and GAPDH were used as loading controls.
(TIF)

Figure S2 LRRK2 protein levels in the Non-Tg and mouse expressing hLRRK2-WT or the R1441G mutation. **A)** Schematic LRRK2 protein showing the different epitopes for all the LRRK2 antibodies used throughout the study. **B)** Representative western blot of total LRRK2 levels in the mouse models using 2 different antibodies (UDD3 and N241A/34). Both antibodies recognize human and mouse LRRK2. The N231B/34 antibody is human specific, and allows the determination of the contribution of human LRRK2, versus the total LRRK2 observed in each mouse models. The presence of high hLRRK2 levels in the mutant mouse suggests that the levels in the R1441G mouse are largely due to the expression of human LRRK2. This is not the case for hLRRK2-WT, where only a faint band can be observed, thus only a small contribution to hLRRK2 levels in this mouse. Expectedly, no human LRRK2 was detected in both the LRRK2 KO and the Non-Tg.
(TIF)

Figure S3 **Co-immunoprecipitation of Ago2 and LRRK2 from mouse brain. A)** Ago2 (2A8) is immunoprecipitated and the efficiency and specificity (mIgG control) of the pull down were observed by western blot. The absence of direct interaction between LRRK2 and Ago2 is shown by western blot, using the MJFF2 antibody. **B)** Reciprocal immunoprecipitation of LRRK2 from mammalian brain. Two LRRK2 antibodies (MJFF2 and UDD3), along with the negative controls, rabbit IgG and LRRK2 KO, were used to immunoprecipitate LRRK2. Ago2 (C34C6) was not pulled down. Of note, the IP in mouse LRRK2 Wt (top panel) gave the same protein profile than the KO. The efficiency and specificity were determined by reprobing the membrane with MJFF2.
(TIF)

Figure S4 Polysomes fractionation on continuous sucrose gradient. **A)** P10 mouse brain was homogenized in the extraction buffer and proteins fractionated on a 10–50% linear gradient. This age was used because of technical limitations with continuous gradients (not shown). However, similar results were obtained for LRRK2 localization between P10 and P30 brains. Protein fractionation profile is shown as the absorbance at 254 nm. **B)** Western blot analyses of protein fractions. FMRP is a marker for polysomes, where Ago2 was mainly found. LRRK2 was not detected in any fractions under these conditions.
(TIF)

Figure S5 Table overview of IPA-generated pathways. **(A, B)** Schematic of network shapes and the potential relationships are shown. **(C)** Upstream analysis of the MAPT network generated by the IPA program. Genes present in this list were misregulated in the LRRK2 KO mice.
(TIF)

Table S1 Total gene changes in LRRK2 mouse models.
(XLS)

Table S2 Fold changes, p-values, accession numbers and oligo sequences of validated genes.
(XLS)

Table S3 Abundance of mRNAs and miRNA according to FDR values.
(XLS)

Table S4 Total miRNA changes in LRRK2 mouse models.
(XLS)

Table S5 Pathway analysis of LRRK2 mice.
(XLS)

## References

[1] Lees AJ, Hardy J, Revesz T (2009) Parkinson’s disease. Lancet 373: 2055–2066. 19524782 10.1016/S0140-6736(09)60492-X

[2] Dickson DW (2012) Parkinson’s disease and parkinsonism: neuropathology. Cold Spring Harb Perspect Med 2. 10.1101/cshperspect.a009258 PMC3405828 22908195

[3] Funayama M, Hasegawa K, Ohta E, Kawashima N, Komiyama M, et al (2005) An LRRK2 mutation as a cause for the parkinsonism in the original PARK8 family. Ann Neurol 57: 918–921. 15880653 10.1002/ana.20484

[4] Paisan-Ruiz C, Jain S, Evans EW, Gilks WP, Simon J, et al (2004) Cloning of the gene containing mutations that cause PARK8-linked Parkinson’s disease. Neuron 44: 595–600. 15541308 10.1016/j.neuron.2004.10.023

[5] Cookson MR (2010) The role of leucine-rich repeat kinase 2 (LRRK2) in Parkinson’s disease. Nat Rev Neurosci 11: 791–797. 21088684 10.1038/nrn2935 PMC4662256

[6] MacLeod D, Dowman J, Hammond R, Leete T, Inoue K, et al (2006) The familial Parkinsonism gene LRRK2 regulates neurite process morphology. Neuron 52: 587–593. 17114044 10.1016/j.neuron.2006.10.008

[7] Matta S, Van Kolen K, da Cunha R, van den Bogaart G, Mandemakers W, et al (2012) LRRK2 controls an EndoA phosphorylation cycle in synaptic endocytosis. Neuron 75: 1008–1021. 22998870 10.1016/j.neuron.2012.08.022

[8] Ferree A, Guillily M, Li H, Smith K, Takashima A, et al (2012) Regulation of physiologic actions of LRRK2: focus on autophagy. Neurodegener Dis 10: 238–241. 22204929 10.1159/000332599 PMC3363354

[9] Dorval V, Hebert SS (2012) LRRK2 in Transcription and Translation Regulation: Relevance for Parkinson’s Disease. Front Neurol 3: 12. 22363314 10.3389/fneur.2012.00012 PMC3276974

[10] Habig K, Walter M, Poths S, Riess O, Bonin M (2008) RNA interference of LRRK2-microarray expression analysis of a Parkinson’s disease key player. Neurogenetics 9: 83–94. 18097693 10.1007/s10048-007-0114-0

[11] Schulz C, Paus M, Frey K, Schmid R, Kohl Z, et al (2011) Leucine-rich repeat kinase 2 modulates retinoic acid-induced neuronal differentiation of murine embryonic stem cells. PLoS ONE 6: e20820. 21695257 10.1371/journal.pone.0020820 PMC3111438

[12] Nikonova EV, Xiong Y, Tanis KQ, Dawson VL, Vogel RL, et al (2012) Transcriptional responses to loss or gain of function of the leucine-rich repeat kinase 2 (LRRK2) gene uncover biological processes modulated by LRRK2 activity. Hum Mol Genet 21: 163–174. 21972245 10.1093/hmg/ddr451 PMC3235012

[13] Devine MJ, Kaganovich A, Ryten M, Mamais A, Trabzuni D, et al (2011) Pathogenic LRRK2 mutations do not alter gene expression in cell model systems or human brain tissue. PLoS ONE 6: e22489. 21799870 10.1371/journal.pone.0022489 PMC3142158

[14] Meister G (2013) Argonaute proteins: functional insights and emerging roles. Nat Rev Genet. 10.1038/nrg3462 23732335

[15] Dachsel JC, Taylor JP, Mok SS, Ross OA, Hinkle KM, et al (2007) Identification of potential protein interactors of Lrrk2. Parkinsonism Relat Disord 13: 382–385. 17400507 10.1016/j.parkreldis.2007.01.008 PMC2970619

[16] Gehrke S, Imai Y, Sokol N, Lu B (2010) Pathogenic LRRK2 negatively regulates microRNA-mediated translational repression. Nature 466: 637–641. 20671708 10.1038/nature09191 PMC3049892

[17] Baek D, Villen J, Shin C, Camargo FD, Gygi SP, et al (2008) The impact of microRNAs on protein output. Nature 455: 64–71. 18668037 10.1038/nature07242 PMC2745094

[18] Selbach M, Schwanhausser B, Thierfelder N, Fang Z, Khanin R, et al (2008) Widespread changes in protein synthesis induced by microRNAs. Nature 455: 58–63. 18668040 10.1038/nature07228

[19] Djuranovic S, Nahvi A, Green R (2012) miRNA-mediated gene silencing by translational repression followed by mRNA deadenylation and decay. Science 336: 237–240. 22499947 10.1126/science.1215691 PMC3971879

[20] Hu W, Coller J (2012) What comes first: translational repression or mRNA degradation? The deepening mystery of microRNA function. Cell Res 22: 1322–1324. 22613951 10.1038/cr.2012.80 PMC3434348

[21] Westerlund M, Belin AC, Anvret A, Bickford P, Olson L, et al (2008) Developmental regulation of leucine-rich repeat kinase 1 and 2 expression in the brain and other rodent and human organs: Implications for Parkinson’s disease. Neuroscience 152: 429–436. 18272292 10.1016/j.neuroscience.2007.10.062

[22] Li Y, Liu W, Oo TF, Wang L, Tang Y, et al (2009) Mutant LRRK2(R1441G) BAC transgenic mice recapitulate cardinal features of Parkinson’s disease. Nat Neurosci 12: 826–828. 19503083 10.1038/nn.2349 PMC2845930

[23] Li T, Yang D, Sushchky S, Liu Z, Smith WW (2011) Models for LRRK2-Linked Parkinsonism. Parkinsons Dis 2011: 942412. 21603132 10.4061/2011/942412 PMC3096154

[24] Baptista MA, Dave KD, Sheth NP, De Silva SN, Carlson KM, et al (2013) A strategy for the generation, characterization and distribution of animal models by The Michael J. Fox Foundation for Parkinson’s Research. Dis Model Mech 6: 1316–1324. 24046356 10.1242/dmm.011940 PMC3820256

[25] Mandemakers W, Snellinx A, O’Neill MJ, de Strooper B (2012) LRRK2 expression is enriched in the striosomal compartment of mouse striatum. Neurobiol Dis 48: 582–593. 22850484 10.1016/j.nbd.2012.07.017

[26] Davies P, Hinkle KM, Sukar NN, Sepulveda B, Mesias R, et al (2013) Comprehensive characterization and optimization of anti-LRRK2 (leucine-rich repeat kinase 2) monoclonal antibodies. Biochem J 453: 101–113. 23560750 10.1042/BJ20121742 PMC3682752

[27] Granic A, Potter H (2013) Mitotic spindle defects and chromosome mis-segregation induced by LDL/cholesterol-implications for Niemann-Pick C1, Alzheimer’s disease, and atherosclerosis. PLoS ONE 8: e60718. 23593294 10.1371/journal.pone.0060718 PMC3625184

[28] Wang X, Yan MH, Fujioka H, Liu J, Wilson-Delfosse A, et al (2012) LRRK2 regulates mitochondrial dynamics and function through direct interaction with DLP1. Hum Mol Genet 21: 1931–1944. 22228096 10.1093/hmg/dds003 PMC3315202

[29] Bailey RM, Covy JP, Melrose HL, Rousseau L, Watkinson R, et al. (2013) LRRK2 phosphorylates novel tau epitopes and promotes tauopathy. Acta Neuropathol. 10.1007/s00401-013-1188-4 PMC3830748 24113872

[30] Kawakami F, Shimada N, Ohta E, Kagiya G, Kawashima R, et al. (2013) LRRK2 regulates tau phosphorylation through direct activation of GSK-3beta. FEBS J. 10.1111/febs.12579 24165324

[31] Dorval V, Smith PY, Delay C, Calvo E, Planel E, et al (2012) Gene network and pathway analysis of mice with conditional ablation of dicer in post-mitotic neurons. PLoS ONE 7: e44060. 22952873 10.1371/journal.pone.0044060 PMC3428293

[32] Hebert SS, Papadopoulou AS, Smith P, Galas MC, Planel E, et al (2010) Genetic ablation of Dicer in adult forebrain neurons results in abnormal tau hyperphosphorylation and neurodegeneration. Hum Mol Genet 19: 3959–3969. 20660113 10.1093/hmg/ddq311

[33] Cimmino A, Calin GA, Fabbri M, Iorio MV, Ferracin M, et al (2005) miR-15 and miR-16 induce apoptosis by targeting BCL2. Proc Natl Acad Sci U S A 102: 13944–13949. 16166262 10.1073/pnas.0506654102 PMC1236577

[34] Bandmann O, Cookson MR (2012) Parkinson disease, cancer, and LRRK2: causation or association? Neurology 78: 772–773. 22323745 10.1212/WNL.0b013e318249f744

[35] Nelson PT, Hatzigeorgiou AG, Mourelatos Z (2004) miRNP:mRNA association in polyribosomes in a human neuronal cell line. RNA 10: 387–394. 14970384 10.1261/rna.5181104 PMC1370934

[36] Molotski N, Soen Y (2012) Differential association of microRNAs with polysomes reflects distinct strengths of interactions with their mRNA targets. RNA 18: 1612–1623. 22836355 10.1261/rna.033142.112 PMC3425777

[37] Khandjian EW, Huot ME, Tremblay S, Davidovic L, Mazroui R, et al (2004) Biochemical evidence for the association of fragile X mental retardation protein with brain polyribosomal ribonucleoparticles. Proc Natl Acad Sci U S A 101: 13357–13362. 15329415 10.1073/pnas.0405398101 PMC516571

[38] Wang WX, Wilfred BR, Hu Y, Stromberg AJ, Nelson PT (2010) Anti-Argonaute RIP-Chip shows that miRNA transfections alter global patterns of mRNA recruitment to microribonucleoprotein complexes. RNA 16: 394–404. 20042474 10.1261/rna.1905910 PMC2811668

[39] Nadler JJ, Zou F, Huang H, Moy SS, Lauder J, et al (2006) Large-scale gene expression differences across brain regions and inbred strains correlate with a behavioral phenotype. Genetics 174: 1229–1236. 16980393 10.1534/genetics.106.061481 PMC1667050

[40] Baudry A, Mouillet-Richard S, Schneider B, Launay JM, Kellermann O (2010) miR-16 targets the serotonin transporter: a new facet for adaptive responses to antidepressants. Science 329: 1537–1541. 20847275 10.1126/science.1193692

[41] Finnerty JR, Wang W-X, Hébert SS, Wilfred BR, Mao G, et al (2010) The miR-15/107 Group of MicroRNA Genes: Evolutionary Biology, Cellular Functions, and Roles in Human Diseases. Journal of molecular biology 402: 491–509. 20678503 10.1016/j.jmb.2010.07.051 PMC2978331

[42] Nishi H, Ono K, Iwanaga Y, Horie T, Nagao K, et al (2010) MicroRNA-15b modulates cellular ATP levels and degenerates mitochondria via Arl2 in neonatal rat cardiac myocytes. J Biol Chem 285: 4920–4930. 20007690 10.1074/jbc.M109.082610 PMC2836096

[43] Chartier-Harlin MC, Dachsel JC, Vilarino-Guell C, Lincoln SJ, Lepretre F, et al (2011) Translation initiator EIF4G1 mutations in familial Parkinson disease. Am J Hum Genet 89: 398–406. 21907011 10.1016/j.ajhg.2011.08.009 PMC3169825

[44] Trancikova A, Mamais A, Webber PJ, Stafa K, Tsika E, et al (2012) Phosphorylation of 4E-BP1 in the mammalian brain is not altered by LRRK2 expression or pathogenic mutations. PLoS ONE 7: e47784. 23082216 10.1371/journal.pone.0047784 PMC3474772

[45] Gareau C, Houssin E, Martel D, Coudert L, Mellaoui S, et al (2013) Characterization of fragile X mental retardation protein recruitment and dynamics in Drosophila stress granules. PLoS ONE 8: e55342. 23408971 10.1371/journal.pone.0055342 PMC3567066

[46] Smith PY, Delay C, Girard J, Papon MA, Planel E, et al (2011) MicroRNA-132 loss is associated with tau exon 10 inclusion in progressive supranuclear palsy. Hum Mol Genet 20: 4016–4024. 21807765 10.1093/hmg/ddr330

[47] Nelson PT, De Planell-Saguer M, Lamprinaki S, Kiriakidou M, Zhang P, et al (2007) A novel monoclonal antibody against human Argonaute proteins reveals unexpected characteristics of miRNAs in human blood cells. RNA 13: 1787–1792. 17720879 10.1261/rna.646007 PMC1986805

