# Supplementary figures and images for: Gene and MicroRNA Transcriptome Analysis of Parkinson's Related LRRK2 Mouse Models
Source: PLoS One. 2014 Jan 10;9(1):e85510. doi: 10.1371/journal.pone.0085510 (PMC3888428; doi:10.1371/journal.pone.0085510)

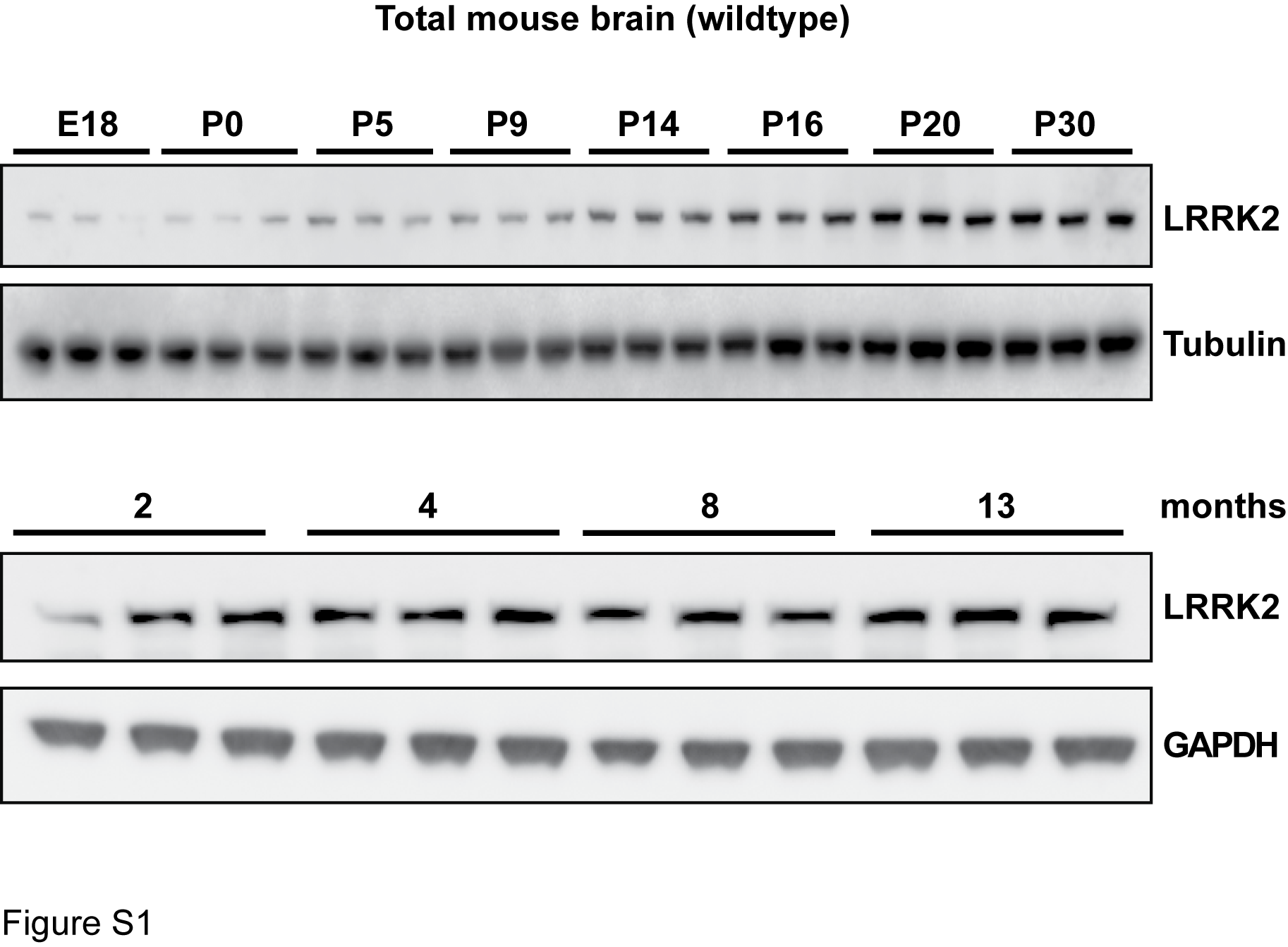

Supplement: Figure S1 — LRRK2 levels during mouse development. Representative western blot analysis of brain LRRK2 expression at different time-points, from embryonic day 18 (E18) to 13 months of age. Tubulin and GAPDH were used as loading controls. (TIF) [file pone.0085510.s001.tif]

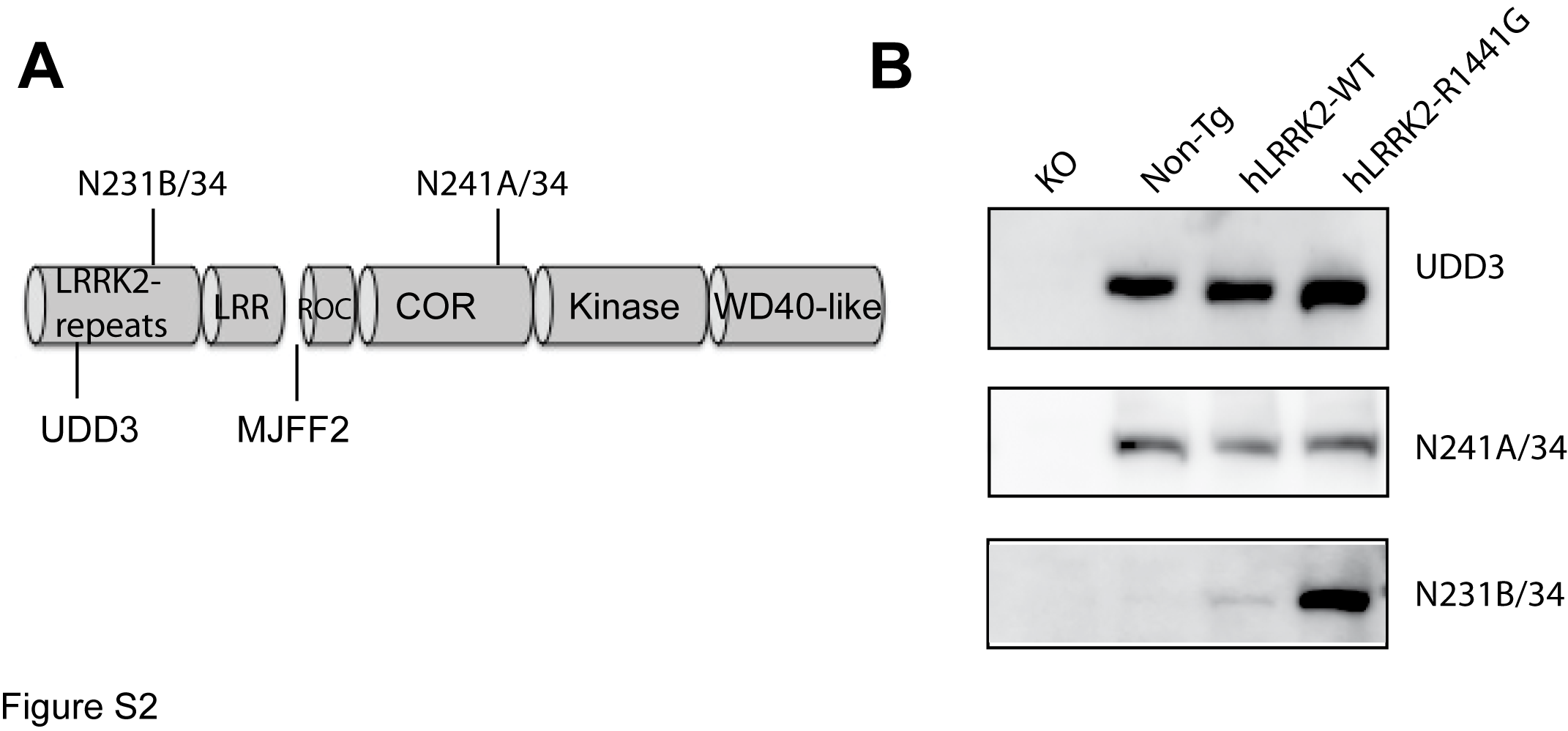

Supplement: Figure S2 — LRRK2 protein levels in the Non-Tg and mouse expressing hLRRK2-WT or the R1441G mutation. A) Schematic LRRK2 protein showing the different epitopes for all the LRRK2 antibodies used throughout the study. B) Representative western blot of total LRRK2 levels in the mouse models using 2 different antibodies (UDD3 and N241A/34). Both antibodies recognize human and mouse LRRK2. The N231B/34 antibody is human specific, and allows the determination of the contribution of human LRRK2, versus the total LRRK2 observed in each mouse models. The presence of high hLRRK2 levels in the mutant mouse suggests that the levels in the R1441G mouse are largely due to the expression of human LRRK2. This is not the case for hLRRK2-WT, where only a faint band can be observed, thus only a small contribution to hLRRK2 levels in this mouse. Expectedly, no human LRRK2 was detected in both the LRRK2 KO and the Non-Tg. (TIF) [file pone.0085510.s002.tif]

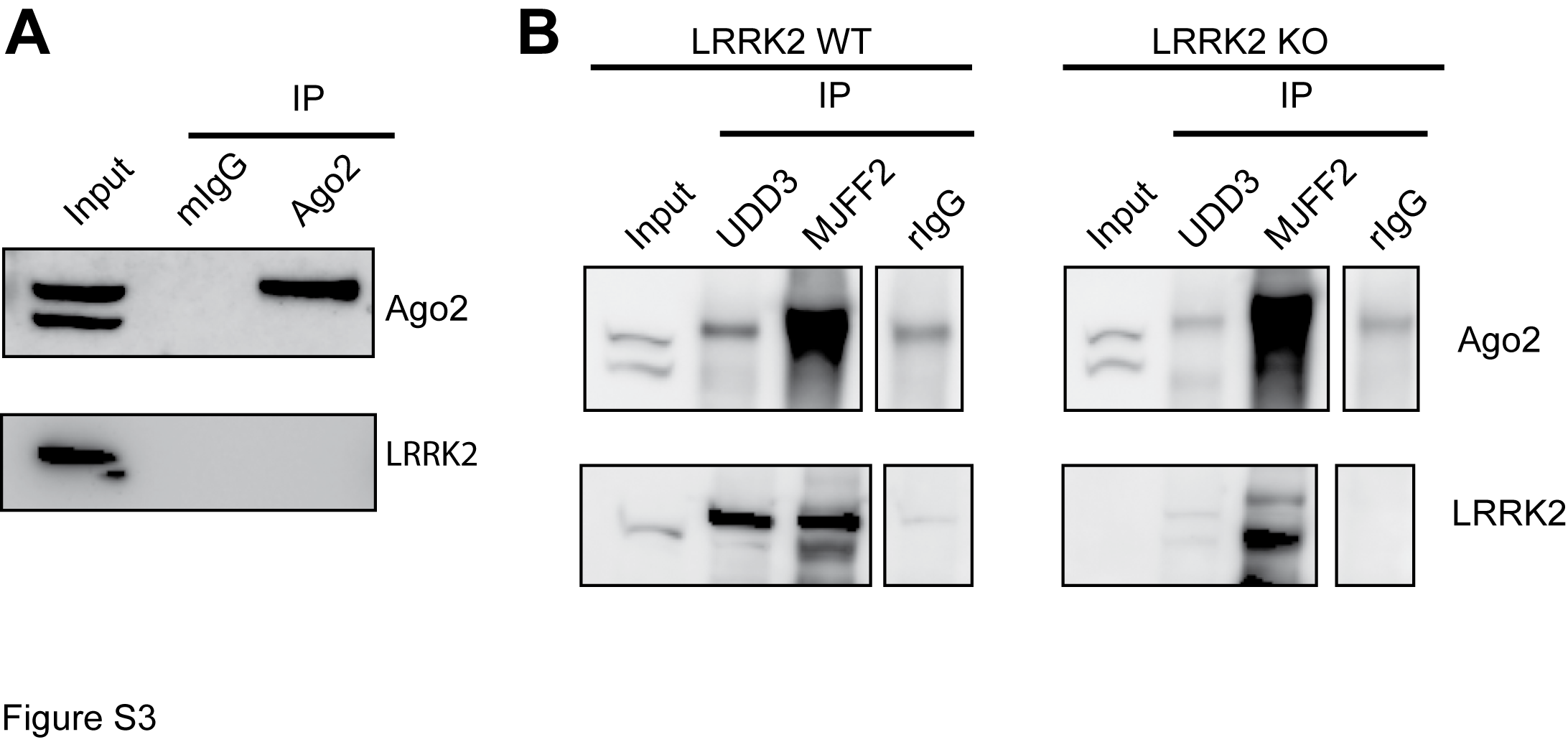

Supplement: Figure S3 — Co-immunoprecipitation of Ago2 and LRRK2 from mouse brain. A) Ago2 (2A8) is immunoprecipitated and the efficiency and specificity (mIgG control) of the pull down were observed by western blot. The absence of direct interaction between LRRK2 and Ago2 is shown by western blot, using the MJFF2 antibody. B) Reciprocal immunoprecipitation of LRRK2 from mammalian brain. Two LRRK2 antibodies (MJFF2 and UDD3), along with the negative controls, rabbit IgG and LRRK2 KO, were used to immunoprecipitate LRRK2. Ago2 (C34C6) was not pulled down. Of note, the IP in mouse LRRK2 Wt (top panel) gave the same protein profile than the KO. The efficiency and specificity were determined by reprobing the membrane with MJFF2. (TIF) [file pone.0085510.s003.tif]

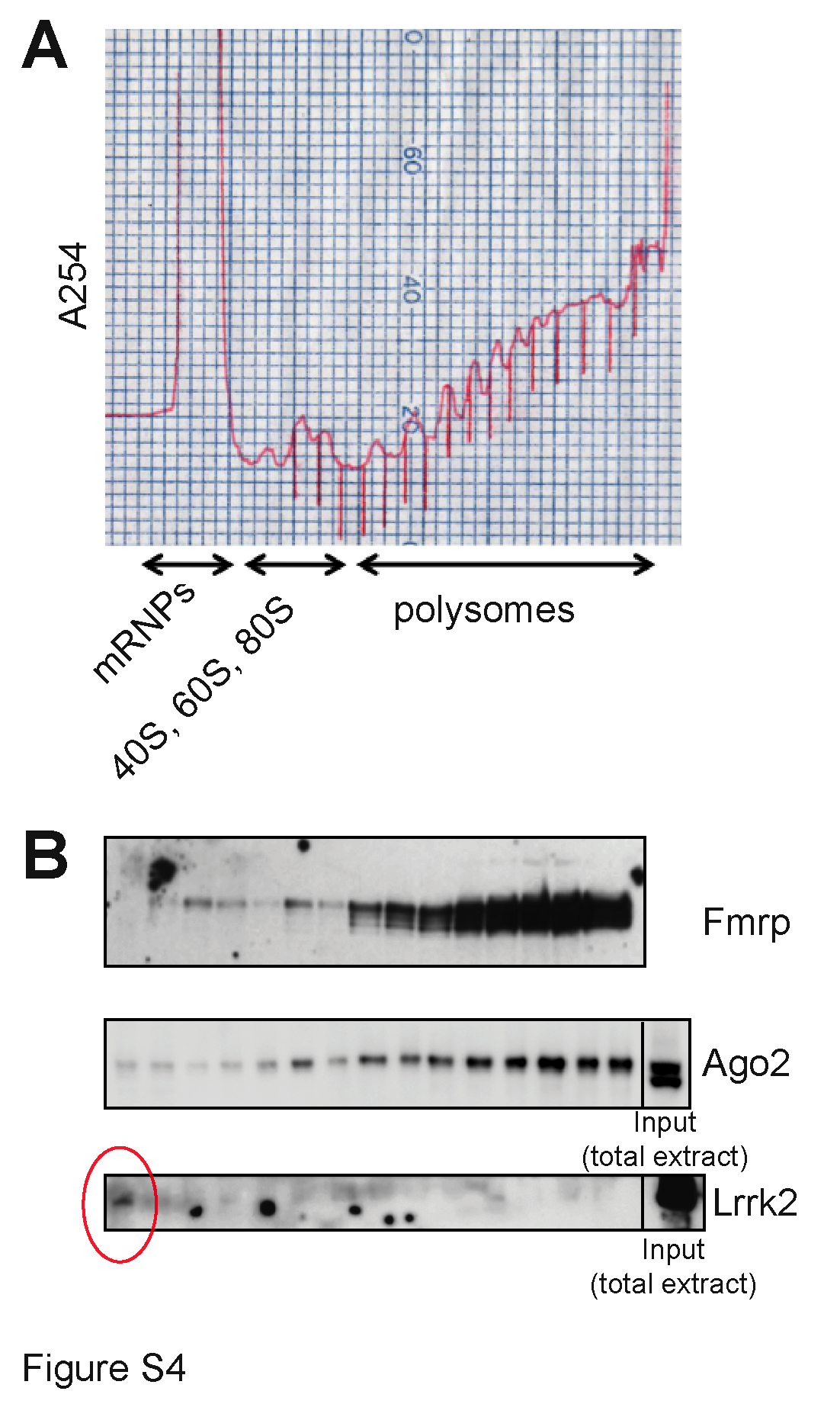

Supplement: Figure S4 — Polysomes fractionation on continuous sucrose gradient. A) P10 mouse brain was homogenized in the extraction buffer and proteins fractionated on a 10–50% linear gradient. This age was used because of technical limitations with continuous gradients (not shown). However, similar results were obtained for LRRK2 localization between P10 and P30 brains. Protein fractionation profile is shown as the absorbance at 254 nm. B) Western blot analyses of protein fractions. FMRP is a marker for polysomes, where Ago2 was mainly found. LRRK2 was not detected in any fractions under these conditions. (TIF) [file pone.0085510.s004.tif]

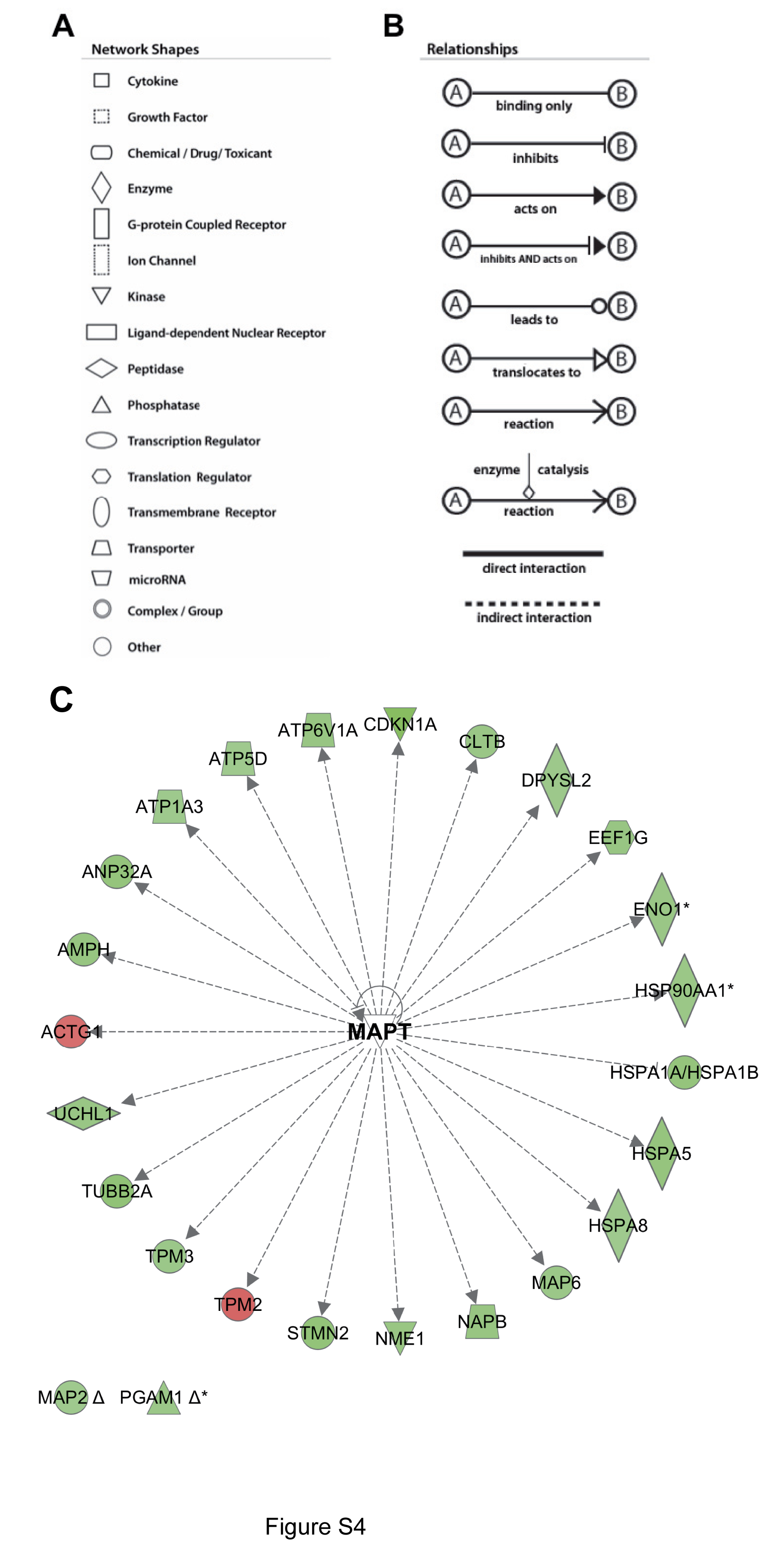

Supplement: Figure S5 — Table overview of IPA-generated pathways. (A, B) Schematic of network shapes and the potential relationships are shown. (C) Upstream analysis of the MAPT network generated by the IPA program. Genes present in this list were misregulated in the LRRK2 KO mice. (TIF) [file pone.0085510.s005.tif]
